# Derazantinib (ARQ 087) in advanced or inoperable FGFR2 gene fusion-positive intrahepatic cholangiocarcinoma

**DOI:** 10.1038/s41416-018-0334-0

**Published:** 2018-11-13

**Authors:** Vincenzo Mazzaferro, Bassel F. El-Rayes, Michele Droz dit Busset, Christian Cotsoglou, William P. Harris, Nevena Damjanov, Gianluca Masi, Lorenza Rimassa, Nicola Personeni, Fadi Braiteh, Vittorina Zagonel, Kyriakos P. Papadopoulos, Terence Hall, Yunxia Wang, Brian Schwartz, Julia Kazakin, Sherrie Bhoori, Filippo de Braud, Walid L. Shaib

**Affiliations:** 1 Department of Surgery and Oncology, Istituto Nazionale Tumori IRCCS, Milan, Italy; 2 University of Milan, Milan, Italy; 3 Winship Cancer Institute, Emory University, Atlanta, GA USA; 4 Seattle Cancer Care Alliance, Seattle, WA USA; 5 Abramson Cancer Center, University of Pennsylvania, Philadelphia, PA USA; 6 Department of Oncology, Pisa University Hospital, Pisa, Italy; 7 Humanitas Cancer Center, Humanitas Clinical and Research Center, Rozzano, Milan, Italy; 8 Department of Medical Biosciences, Humanitas University, Pieve Emanuele, Milan, Italy; 9 Medical Oncology, Comprehensive Cancer Centers of Nevada, Las Vegas, NV USA; 10 Veneto Institute of Oncology IOV - IRCCS, Padua, Italy; 11 South Texas Accelerated Research Therapeutics, San Antonio, TX USA; 12 ArQule, Inc, Burlington, MA USA

**Keywords:** Bile duct cancer, Targeted therapies

## Abstract

**Background:**

Next-generation sequencing has identified actionable genetic aberrations in intrahepatic cholangiocarcinomas (iCCA), including the fibroblast growth factor receptor 2 (FGFR2) fusions. Derazantinib (ARQ 087), an orally bioavailable, multi-kinase inhibitor with potent pan-FGFR activity, has shown preliminary therapeutic activity against FGFR2 fusion-positive iCCA.

**Methods:**

This multicentre, phase 1/2, open-label study enrolled adult patients with unresectable iCCA with FGFR2 fusion, who progressed, were intolerant or not eligible to first-line chemotherapy (NCT01752920). Subjects received derazantinib in continuous daily doses. Tumour response was assessed according to RECIST 1.1 every 8 weeks.

**Results:**

Twenty-nine patients (18 women/11 men; median age, 58.7 years), 2 treatment-naive and 27 who progressed after at least one prior systemic therapy, were enrolled. Overall response rate was 20.7%, disease control rate was 82.8%. Estimated median progression-free survival was 5.7 months (95% CI: 4.04–9.2 months). Treatment-related adverse events (AE) were observed in 27 patients (93.1%, all grades), including asthenia/fatigue (69.0%), eye toxicity (41.4%), and hyperphosphatemia (75.9%). Grade ≥ 3 AEs occurred in 8 patients (27.6%).

**Conclusion:**

Derazantinib demonstrated encouraging anti-tumour activity and a manageable safety profile in patients with advanced, unresectable iCCA with FGFR2 fusion who progressed after chemotherapy. A pivotal trial of derazantinib in iCCA is ongoing (NCT03230318).

## Introduction

Cholangiocarcinomas represent a heterogeneous group of epithelial malignancies originating from the biliary system. They are classified either as intrahepatic (arising proximally to the second order bile ducts), peri-hilar (arising from the tract between the first order bile ducts and the main bile duct, proximally to the cystic duct), or extrahepatic (arising from the distal main bile duct). Intrahepatic cholangiocarcinomas (iCCA) arising within the liver account for ~20% of all cholangiocarcinomas.^1^ Compared to other cholangiocarcinomas, iCCAs are more heterogeneous, differing one from another in regards to their molecular and cell biological characteristics, as well as resistance to treatment.^1–3^

Most patients with iCCA present with advanced, unresectable, or metastatic disease, which limits the number of available treatment options. The current first-line standard-of-care chemotherapy for patients with advanced biliary tract cancers, including iCCA, is the combination of gemcitabine plus cisplatin or other platinum-derived agents,^4^ with a median survival of less than 1 year.^5–9^ There is no proven effective treatment for patients with advanced biliary cancer who progress on first-line chemotherapy, thus identifying second-line treatment for these patients as a high unmet medical need.^10,11^ High degree of drug resistance is common, most likely reflecting the genetic complexity and cellular heterogeneity of iCCA.^1^ Prominent desmoplastic stroma seen in most tumours is yet another barrier to drug delivery.

Next-generation sequencing of iCCA has identified several potentially actionable therapeutic targets. The most common aberrations are isocitrate dehydrogenase (IDH1/2) mutations^12,13^ and FGFR2 mutations or fusions.^14–18^ FGFR genetic aberrations occur in 10 to 16% of iCCAs, more frequently in younger patients (≤40 years), with a non-significant predilection for female sex.^19^ FGFR2 translocations are usually mutually exclusive of KRAS, IDH1/2, and BRAF mutations. Fusion events are usually present in a very high proportion of tumour cells, and most likely represent a clonal hallmark deriving from an early oncogenic “driver” mutation.^15^ Inhibition of FGFR2 could therefore have a high therapeutic impact.

FGFR2 as an actionable target in iCCA has been tested in a number of clinical trials.^20–25^ FGFR2 genetic aberrations (assessed by NGS or FISH) were found to be more frequent in young and female patients with earlier tumour stage. In surgically resected patients, FGFR aberrations had a longer overall survival (OS) compared to wild-type (WT) patients, even after exclusion of patients treated with FGFR inhibitors.^26,27^ In fact, median OS of patients harbouring a FGFR2 aberration was longer than in WT patients (37 vs. 20 months, respectively), without any difference between FGFR2 fusions compared to other aberrations (e.g. mutations or amplifications). However, progression-free survival (PFS), available only in a subset of patients, did not show a significant difference between patients harbouring an FGFR aberration and the WT ones. One of the plausible explanations of a shorter than expected PFS may be development of recurrent secondary FGFR2 kinase domain mutations that are resistant to FGFR inhibition.^25,28^

Derazantinib (ARQ 087) is an orally bioavailable, potent, ATP-competitive, pan-FGFR inhibitor with strong activity against FGFR2, FGFR1, and FGFR3 kinases.^29^ Derazantinib demonstrated potent inhibition of tumour growth in FGFR pathway-activated models, including FGFR2-driven tumour xenografts (FGFR2 amplification/fusion, NCI-H716 and SNU-16 xenograft models), and BaF3/FGFR2 murine transfected cell lines.^29^ In the phase 1 part of our study, derazantinib was well tolerated with manageable toxicities in an unselected patient population, and demonstrated single-agent anti-tumour activity in heavily pretreated patients with FGFR genetic alterations.^30^

Recently, an open-label, dose-escalation and signal-finding phase 1/2 study (ARQ 087–101) of derazantinib in subjects with advanced solid tumours with FGFR genetic alterations, including iCCA with FGFR2 gene fusion, was completed. The study explored safety, tolerability, pharmacokinetics, pharmacodynamics, and efficacy of derazantinib, and defined the recommended phase 2 dose (RP2D), based on cumulative safety data on 80 patients, showing a dose-dependent increase in toxicity when dose levels were increased from 250 to 425 mg QD.^30^

Herein, we report the results from the phase 1/2 of the study, which enrolled consecutive patients with FGFR2 gene fusion-positive advanced iCCA who were treated with derazantinib according to the RP2D^30^ (NCT01752920).

## Methods

### Study design and treatment

Patients were enrolled from August 2014 through January 2017. Two patients were treated with 400 mg daily (QD), as part of the phase 1 trial of derazantinib, while 27 patients received derazantinib at the recommended phase 2 dose (RP2D) of 300 mg QD. Treatment cycles were continuous 28-day periods without any treatment interruption between cycles. Treatment continued until disease progression, unacceptable toxicity, investigator’s decision, or consent withdrawal. Treatment with derazantinib beyond progression was permitted if, in the opinion of the Investigator, the patient continued to derive a clinical benefit. Dose interruption until resolution of toxicity, and a maximum of two dose reductions (to lower dose levels of 200 or 100 mg QD, according to the grade of toxicity), were allowed.

### Patients

Patients with histologically confirmed unresectable or metastatic iCCA and FGFR2 gene fusion confirmed by NGS or fluorescence in situ hybridisation (FISH),^31^ who had progression after at least one prior systemic therapy or were treatment naive but were not eligible for standard first-line chemotherapy, were screened for enrolment. Other detailed eligibility criteria are provided in the Data Supplement. FGFR2 fusion could be determined either locally or centrally in a CLIA-certified facility.

### Endpoints and response assessments

The primary endpoint was safety and tolerability of derazantinib in patients with advanced or unresectable FGFR2 fusion-positive iCCA. Efficacy endpoints included PFS and OS. Other secondary endpoints were overall response rate (RR; complete response [CR] + partial response [PR]) and disease control rate (DCR; CR + PR + stable disease [SD]), assessed by independent local staff radiologists, using computed tomography (CT) or magnetic resonance imaging (MRI). Radiologic response assessments were conducted at baseline and approximately every 8 weeks thereafter according to Response Evaluation Criteria in Solid Tumours (RECIST) version (v) 1.1.^32^

Safety assessments included monitoring of adverse events (AEs), vital signs, haematologic, and clinical biochemistry values weekly during the first cycle and every 2 weeks thereafter. Adverse events were graded using Common Terminology Criteria for Adverse Events (CTCAE) version 4.03, except hyperphosphatemia, not defined by CTCAE, which was graded per ArQule’s criteria as follows: grade 1 (low) was for phosphate levels above the upper limit of normal (ULN) up to 1.2 ULN, not requiring intervention; grade 2 (intermediate) was for phosphate levels above 1.2 ULN up to 1.4 ULN, rated as significant and requiring medical non-invasive intervention; grade 3 (severe) was for hyperphosphatemia from 1.4 ULN to 1.6 ULN, requiring medical treatment and nephrology consultation; grade 4 (life-threatening) was for phosphate above 1.6 ULN, requiring urgent invasive intervention (e.g. haemodialyses).

### Exploratory biomarker analysis

Blood samples were collected for evaluation of soluble pharmacodynamic markers such as serum phosphate and plasma FGF19, 21, and 23. Samples were collected pre-dose on day 1 of cycles 1–6. FGF19, 21, and 23 were measured at ArQule using commercially available ELISA kits (EMD Millipore, Billerica, MA; R&D systems, Minneapolis, MN). Pharmacodynamic parameters including maximum observed response value (Rmax), maximum change from baseline response value (BRmax), BRmax maximum percent change from baseline (B) response value (%BRmax), average percentage difference from baseline on day 1 cycle 2 (%DiffC2D1), and overall average percentage difference, (%DiffMax) were computed using Prism 5 and Excel.

### Statistical analysis

Descriptive statistics were used for the analyses of the demographics, safety and anti-tumour activity data. Patients who received at least one dose of derazantinib were considered evaluable for safety analyses. Patients who received at least one cycle of derazantinib and had at least one disease assessment following the initiation of therapy were considered evaluable for response. PFS was calculated from the date of the first dose to the date of progression or death, and OS from the first dose to death, using the Kaplan–Meier method. Descriptive statistics, tables, plots for safety and efficacy analysis were generated using SAS (version 9.4). The linearity assessment of pharmacodynamic results (by power model) was generated using R (version 3.4.0).

The study protocol was conducted in accordance with Guidelines for Good Clinical Practice, following applicable local regulations and the ethical principles of the Declaration of Helsinki. This study is registered with ClinicalTrials.gov, number NCT01752920.

## Results

### Patients and treatment

Twenty-nine patients (18 women, 11 men, median age 58.7 years, 100% Caucasian), with iCCA and FGFR2 gene fusion, whose cancer was unresectable, were enrolled and treated with derazantinib at eight sites in the United States and Italy between August 2014 and October 2017. Demographics and baseline characteristics of the patients are summarised in Table 1. The iCCA was at an advanced stage, AJCC stage III or IV, in 22 of the 29 (75.9%) patients, with 18 patients (62%) presenting with metastatic disease (stage IV). Two patients were treatment-naive (6.9%) and 27 (93.1%) patients received at least one prior systemic therapy (48.3% had received at least two prior regimens). The median time from the end of the last chemotherapy regimen to cycle 1 day 1 (C1D1) of derazantinib was 2.1 months (range: 0.5–23.5). All patients had measurable disease, and *FGFR2* gene fusion status was confirmed in all enrolled patients (15 patients by FISH and 14 patients by NGS). The median follow-up was 6.7 months (range: 2.9–19.4). At the time of the data cut-off, two patients (6.9%) were still on treatment and 27 (93.1%) had discontinued treatment; the main reasons for discontinuation were radiologically confirmed disease progression (15 patients, 51.7%), adverse events (4 patients, 13.8%), and clinical deterioration (4 patients, 13.8%).

**Table 1:** Baseline patient demographic and clinical characteristics

Characteristics	Patients (*N* = 29) *n* (%)
Median age, years (range)	58.7 (37.9–82.0)
Sex, *n* (%)	
Female	18 (62.1)
Male	11 (37.9)
Race	
White	29 (100.0)
ECOG performance status	
0	19 (65.5)
1	9 (31.0)
2	1 (3.4)
Median time since initial diagnosis, months (range)	14.1 (1.1–76.5)
Tumour stage at study entry (AJCC Cancer Staging Manual, 7th ed.)	
I	1 (3.4)
II	6 (20.7)
III	4 (13.8)
IV	18 (62.0)
Histology	
Well differentiated	3 (10.3)
Moderately differentiated	12 (41.4)
Poorly differentiated/undifferentiated	5 (17.2)
Unspecified	9 (31.0)
Prior systemic regimens	
None	2 (6.9)
1	13 (44.8)
2	10 (34.5)
3	2 (6.9)
4	2 (6.9)
Best response to prior systemic therapy	
Partial response (PR)	4 (13.8)
Stable disease (SD)	9 (31.0)
Complete response (CR)	11 (37.9)
Unknown/not applicable	3 (10.3)
No prior systemic therapy	2 (6.9)
Prior surgery	
No	15 (51.7)
Yes	14 (48.3)
Prior radiation therapy	
No	26 (89.7)
Yes	3 (10.3)

### Efficacy

Treatment efficacy was evaluated in all 29 patients per RECIST v1.1 (Table 2). None of the patients achieved CR. Six patients (20.7%) achieved PR. Eighteen patients (62.1%) had best response of SD and five patients (17.2%) had progressive disease (PD) as their best response.

**Table 2:** Response to treatment with derazantinib (ARQ 087) in 29 patients with advanced or inoperable *FGFR2* gene fusion-positive iCCA

Response	(*N* = 29) *n* (%)
Best response	
Complete response (CR)	0
Partial response (PR)	6 (20.7)
Stable disease (SD)	18 (62.1)
Progressive disease (PD)	5 (17.2)
Overall response rate (PR)	6 (20.7)
Median duration of PR, months	4.6 (95% CI: 2.3–8.9)
Disease control rate (PR + SD)	24 (82.8)
Median duration of disease control, months	5.8 (95% CI: 5.3–8.4)
PFS events	
Progression	22
Death	2
Censored	5
Median PFS, months	5.7 (95% CI: 4.0–9.2)
Median duration of exposure^a^, months (range)	5.6 (1.5–18.2)
Partial response (*N* = 6)	7.9 (5.5–18.2)
Stable disease (*N* = 18)	5.6 (1.5–18.0)
Progressive disease (*N* = 5)	1.8 (1.8–2.5)

Assessed by the investigators as per Response Evaluation Criteria in Solid Tumours v1.1

^a^Duration of exposure in days = last dosing date—first dosing date + 1

**Fig. 1:**
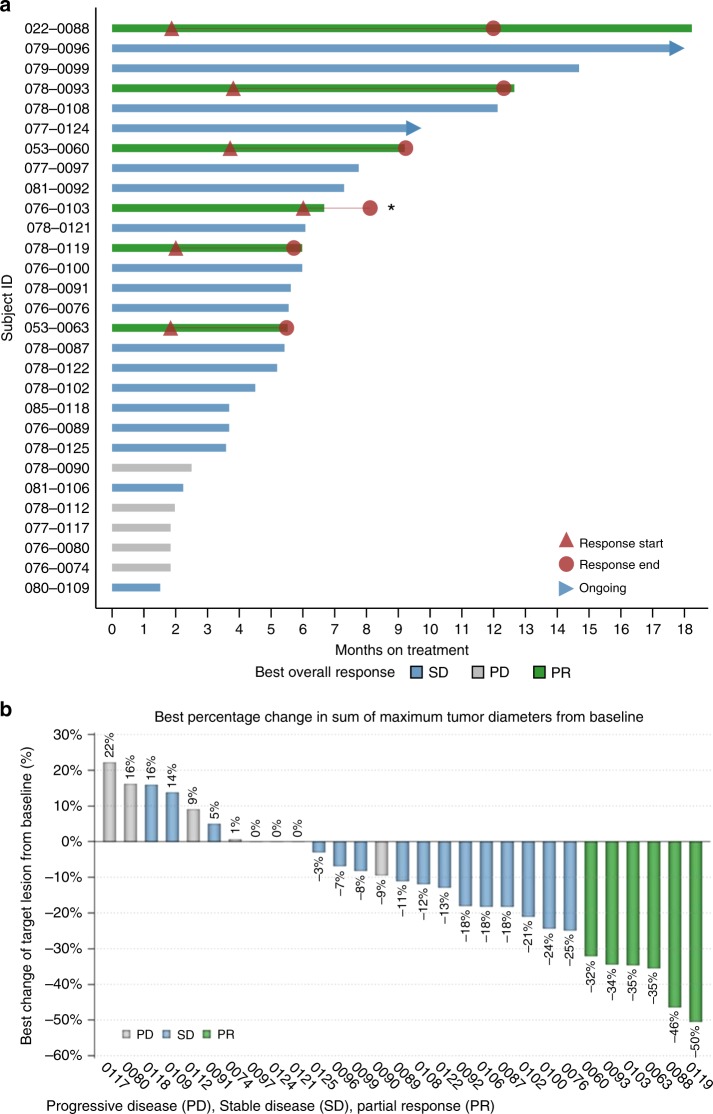
Duration of exposure and best change from baseline. **a** Duration of exposure: swimmer plot presenting duration of exposure, dosing history, and efficacy assessments. **b** Best percentage change in sum of maximum tumour diameters from baseline

The median duration of drug exposure for all patients was 5.6 months with a range from 1.5 to 18.2 months. The ORR was 20.7% with median duration of response of 4.6 months (95% CI: 2.3–8.9 months), and the DCR was 82.8%. The median duration of disease control among the 24 patients who achieved a best overall response of SD or PR was 5.8 months (95% CI: 5.3–8.4 months) (Fig. 1a). The degree of tumour response is reflected in the percent dimensional reduction of the target lesion from baseline (Fig. 1b), for which 19 patients (65.5%) showed some tumour regression.

**Fig. 2:**
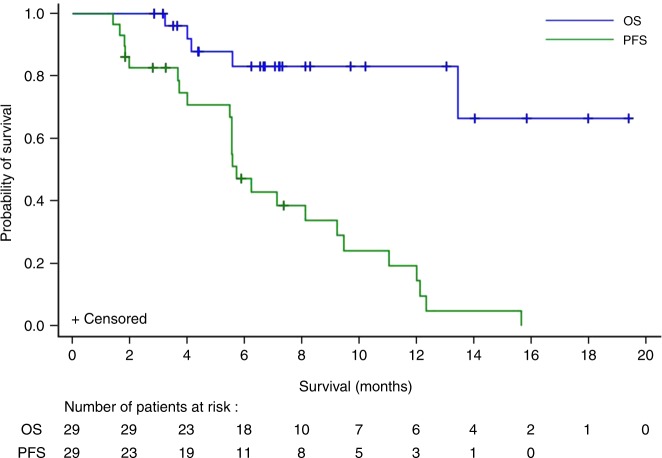
Progression-free survival and overall survival (Kaplan–Meier plots) in 29 patients with advanced or unresectable iCCA treated with derazantinib

**Fig. 3:**
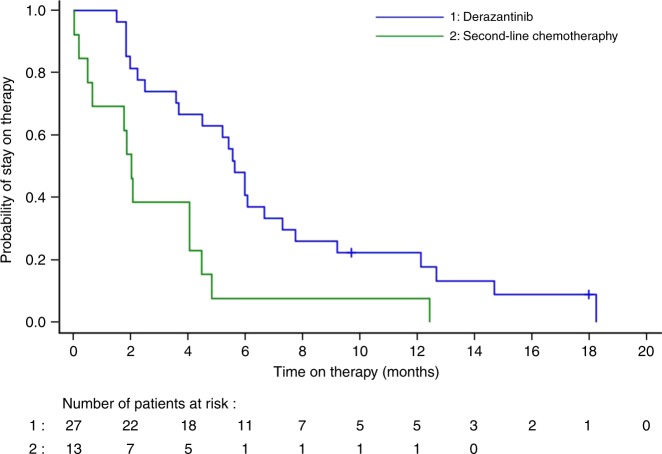
Time on treatment: intra-patient comparison of the second-line chemotherapy regimens vs. derazantinib

As of data cut-off date, the median PFS was 5.7 months (95% CI: 4.04–9.2 months) (Fig. 2). Twenty-four of the 29 patients had a PFS event (2 deaths and 22 patients whose disease progressed). Median OS was not reached after a median follow-up of 20 months (Fig. 2). In 27 patients who received prior systemic therapy, we compared intra-patient time on previous lines of therapy to the time on therapy with derazantinib. Median time on treatment with derazantinib [5.6 months (95% CI: 3.6–7.3 months)] significantly outperformed that obtained in those 13 patients who had received a second-line therapy after progression on the first-line treatment [2 months (95% CI: 0.5–4.0 months, *p* = 0.0019)] (Fig. 3). In the second-line setting, 5 out of 13 patients had received a platinum-based combination, 3 a gemcitabine-based regimen and 3 were treated with capecitabine. In the 27 patients evaluated, time on derazantinib was not significantly prolonged compared to time on treatment with first-line chemotherapy [4.2 months (95% CI: 3.1–5.8 months, *p* = 0.0665)].

Finally, there was no difference in response rate observed in patients who were tested FGFR2 fusion-positive by either test; among 15 FISH-tested patients the best response was PR (*n* = 3), SD (*n* = 10), and PD (n = 2), and among 14 NGS-tested patients was PR (*n* = 3), SD (*n* = 8), and PD (*n* = 3) (Table 1S).

### Treatment safety

Treatment-emergent AEs are reported in data supplement Table 2S. Twenty-seven (93.1%) patients experienced at least one study drug-related AE. The most common AEs were dry mouth and nausea (44.8%); asthenia, fatigue (34.5%); dysgeusia, vomiting (31%); alopecia, blurred vision (24.1%); ALT increase, diarrhoea (20.7%); AST increase, decreased appetite (17.2%). Grade 3 and 4 treatment-related AEs were observed in 8 patients (27.6%), including 1 serious AE (SAE) of treatment-unrelated upper gastrointestinal haemorrhage. There were five treatment-related AEs (asthenia, corneal erosion, diplopia, dry eye, and upper gastrointestinal haemorrhage) in 4 patients (13.8%) that led to treatment discontinuation. Two deaths occurred during the follow-up period; none were attributed to derazantinib.

Hyperphosphatemia was reported in 22 (75.9%) patients, including grade 3 in 3 (10.3%) patients. No dose interruption or modification of derazantinib treatment was required for hyperphosphatemia.

Eye toxicity was reported in 12 (41.4%) patients with 2 (6.9%) patients experiencing grade 3 events (dry eye, blurred vision). Study treatment was temporarily interrupted in 4 (13.8%) patients and dose reduced in 3 (10.3%) patients. Treatment was required in 5 (17.2%) patients with blepharitis, corneal disorder, corneal erosion, diplopia, dry eye, keratitis, and blurred vision. Dose interruption and/or reduction was required in 7 patients (24.1%) to manage derazantinib-related toxicity. Complete dose reduction and interruption data are described in Table 3S. The median time to occurrence of first AE was 53 days for fatigue-asthenia, 15 for hyperphosphatemia, 22 for liver function tests increase, and 50 days for ocular toxicity.

### Pharmacodynamic results

FGF parameters and phosphate level at baseline with percent changes during the follow-up are summarised in data supplement Table 4S. Preliminary analysis showed changes in serum phosphate and FGFs. Overall, mean serum phosphate level increased by an average of 1.2 mg/dL over baseline, while FGF19 and FGF23 showed mean increases in concentrations of 361 and 140 pg/mL respectively. FGF21 mean concentration decreased by 54 pg/mL. All four biomarkers showed a mean percentage change from baseline ranging from 5.6 to 208% on cycle 2 day1.

## Discussion

In our series of 29 patients with advanced or unresectable FGFR2 gene fusion-positive iCCA, derazantinib demonstrated promising efficacy data with a median PFS of 5.7 months (95% CI: 4.04–9.2 months), ORR of 20.7% with median duration of response of 4.6 months (95% CI: 2.3–8.9 months), DCR of 82.8% with median duration of 5.8 months (95% CI: 5.3–8.4 months), and a predictable and manageable safety profile. Median OS could not be estimated due to too few events occurring during the study period. However, the 95% CI lower boundary of OS was estimated to be 13.4 months. Also supporting the positive impact of derazantinib on patient outcome was the longer time on derazantinib observed in the 27 patients who received prior systemic treatment with respect to the time on therapy achieved with second-line chemotherapy (median 5.6 vs. 2 months, respectively, *p* = 0.0019).

Commonly observed AEs included asthenia/fatigue, hyperphosphatemia, eye toxicity and increase in ALT/AST, similar to safety profiles observed with other pan-FGFR inhibitors, such as BGJ398 and JNJ-42756493.^20,23,27^ As expected, hyperphosphatemia was part of the observed treatment toxicity, as it is an on-target treatment effect, associated with FGFR inhibition mediated by FGF23 signalling through FGFR1.^33,34^ Although hyperphosphatemia has been observed in this selected patient population at a higher rate compared to the unselected patient population treated with derazantinib (75.9% vs. 51.3%), the effect was modest, no chelation therapy was required, and most patients continued treatment with derazantinib at full dose.

Eye toxicity was mostly mild to moderate in severity, with only 2 grade 3 events that resolved after treatment modification or discontinuation; it included blurred vision, conjunctivitis, dry eye and reduced visual acuity. Possible explanation of eye toxicity might be the role played by FGFR2 in cornea development.^35^ Overall, to manage derazantinib-related eye toxicity, dose interruption and/or reduction was required in 7 patients (24.1%).

Our results compare favourably to those achieved in a recently published phase II trial with a different FGFR-selective small molecule kinase inhibitor (SMKI) – BGJ398 – administered on a 28-day cycle (3 weeks on and 1 week off) in iCCA with FGFR2 gene aberration who progressed on or were intolerant to first-line therapy. Such study of 61 patients reported an ORR of 18.8%, a DCR of 75.4% and PFS of 5.8 months in the 48 FGFR2 fusion-positive patients, with an OS and ORR that was superior in patients harbouring FGFR2 fusions compared to patients with other FGFR genetic aberrations.^23^

Our study showed an increase in mean percentage change from baseline for FGF23 that is consistent with data reported for other FGFR inhibitors,^23,36^ similar to changes seen with FGF19 and FGF21 that act in an endocrine signalling role, which makes analytical monitoring relatively easy. FGF19, along with FGFR4, is believed to be involved in the progression of hepatocellular carcinoma,^37^ and increased FGF21 levels have been investigated as a potential biomarker for renal cell carcinoma.^38^ These associations with cancer make FGF19 and FGF21 interesting potential biomarkers for FGFR inhibitors; we are not aware of any published data on FGF19 and FGF21 changes in patients treated with FGFR inhibitors. Although our data is of a preliminary nature, we believe that FGF19 may be a useful biomarker. Overall, the observed C2D1 increases in all four analytes support the conclusion that the dose of 300 mg QD disrupts FGFR signal transmission.

Our study has limitations, including a relatively small number of patients, lack of independent radiologic review, lack of central genetic testing, and absence of quality-of-life assessments. The presented results should be framed in the unique clinical phenotype of patients with FGFR2 gene aberrations, exhibiting peculiar pathological (prominent intraductal cancer growth) and clinical features (longer survival).^31^ Although a more indolent disease course of these patients may be attributable to a less aggressive biology, with consequent prolongation of PFS in patients harbouring FGFR2 translocation regardless of targeted therapy, there are no data about PFS in the second-line setting in this cohort. Furthermore, previous evidence showed that PFS did not show significant differences between patients harbouring FGFR aberrations and those with WT FGFR, despite considering the possible development of recurrent secondary FGFR2 kinase domain mutations resistant to FGFR inhibition.^25,28^

Additionally, limitations in the time on treatment analysis with intra-patient comparison between second-line chemotherapy and treatment with derazantinib should be mentioned: a shorter than expected time on treatment with second-line chemotherapy preceding FGFR2-targeted therapy (2 months) may be partially explained by a selection bias of non-responders to treatment with derazantinib. Nonetheless, derazantinib was administered as a third or subsequent line treatment in 14/29 patients (48.3%), thus reinforcing the value of derazantinib compared to second-line chemotherapy in this setting. Overall, ours and other results suggest that anti-FGFR treatment improves the outcome in this iCCA subgroup.^9,28,39^ More data are needed to clarify the outcome of this subpopulation, and pooling of data will be necessary to provide better indications about the prognostic role of FGFR2 aberration.

Despite the good response rate, the shortcoming of such therapy, as with other kinase inhibitors, is the duration of response. Little is known about the mechanisms of secondary resistance. In a recent proof of concept paper, the development of new point mutations in the kinase domain of the FGFR2 receptor was detected during treatment with another FGFR2 inhibitor.^40^ Such mutations may activate the kinase receptor despite the binding of the inhibitor. In this scenario, structurally distinct FGFR inhibitors may overcome specific secondary mutations and pave the way to new approaches for the treatment of patients undergoing disease progression following response to derazantinib.

Finally, the present study supports FISH and NGS use for genetic screening (Table 1S), since no difference in terms of response was observed between patients tested positive by either method. Given the number of the potential fusion partners, we can anticipate that not all “fusions” will be oncogenic drivers and be sensitive to FGFR inhibitors.

In conclusion, derazantinib monotherapy demonstrated a favourable safety profile at a dose of 300 mg daily with promising anti-tumour activity in a selected patient population of iCCA with FGFR2 gene fusion following disease progression on first-line systemic chemotherapy. These promising results have prompted a recently initiated pivotal study in patients with FGFR2 gene fusion-positive iCCA (NCT03230318). In this larger study, time-related endpoints such as PFS, as well as quality-of-life assessments and FGF19 monitoring, will be addressed. Given the peculiar clinical features associated with FGFR2 fusion, possibly affecting patients’ outcomes, future randomised trials are needed to clarify the survival advantage of derazantinib compared to cytotoxic chemotherapy.

## Electronic supplementary material

Data Supplement: tables

## Availability of data and material

Raw data is available upon request. Please contact the corresponding author for details.
