# Supplementary material for: Derazantinib (ARQ 087) in advanced or inoperable FGFR2 gene fusion-positive intrahepatic cholangiocarcinoma
Source: Br J Cancer. 2018 Nov 13;120(2):165–71. doi: 10.1038/s41416-018-0334-0 (PMC6342954; doi:10.1038/s41416-018-0334-0)
Supplement: Supplementary file 1 — Data Supplement: tables [file 41416_2018_334_MOESM1_ESM.docx]

**DATA SUPPLEMENT TABLES**

Table 1S. FGFR2 Fusion Status and Best Response

Table 2S. Adverse events reported as related to derazantinib

Table 3S. Dose reductions and interruptions

Table 4S. Pharmacodynamic results of FGF parameters

**Table 1S.** FGFR2 Fusion Status and Best Response

| FGFR2 fusion status by FISH or NGS and fusion partners | Number of patients and best response |
| --- | --- |
| FISH (PR+SD+PD) | 15 (3PR+10SD+2PD) |
| NGS (PR+SD+PD) | 14 (3PR+8SD+3PD) |
| - FGFR2-BICC1 | 4 (1PR+2SD+1PD) |
| - FGFR2-KIAA1217 | 2 (1PR+1SD) |
| - FGFR2-SH3GLB | 1 (PR) |
| - FGFR2-KIAA1967 | 1 (SD) |
| - FGFR2-AHCYL1 | 1 (SD) |
| - FGFR2-BFSP2 | 1 (SD) |
| - FGFR2-CCDC6 | 1 (SD) |
| - FGFR2-TACC2 | 1 (SD) |
| - FGFR2-CIT | 1 (PD) |
| - FGFR2-TACC1 | 1 (PD) |

**Table 2S.** Adverse events >5% or ≥G3 reported as related to derazantinib

| **Preferred term** | **All Grades n(%)** | **Grade 3 and 4 n(%)** |
| --- | --- | --- |
| Number of all patients (N=29) who experience at least one AE | 27 (93.1) | 8 (27.6) |
| Dry mouth | 13 (44.8) | 0 |
| Nausea | 13 (44.8) | 0 |
| Fatigue | 10 (34.5) | 1 (3.4) |
| Asthenia | 10 (34.5) | 2 (6.9) |
| Dysgeusia | 9 (31.0) | 0 |
| Vomiting | 9 (31.0) | 1 (3.4) |
| Alopecia | 7 (24.1) | 0 |
| Vision blurred | 7 (24.1) | 1 (3.4) |
| Diarrhea | 6 (20.7) | 0 |
| Alanine aminotransferase increased | 6 (20.7) | 1 (3.4) |
| Dry eye | 5 (17.2) | 1 (3.4) |
| Decreased appetite | 5 (17.2) | 0 |
| Aspartate aminotransferase increased | 5 (17.2) | 1 (3.4) |
| Conjunctivitis | 4 (13.8) | 0 |
| Anaemia | 3 (10.3) | 0 |
| Dry skin | 3 (10.3) | 0 |
| Pruritus | 3 (10.3) | 0 |
| Visual acuity reduced | 3 (10.3) | 0 |
| Dizziness | 2 (6.9) | 0 |
| Dermatitis | 2 (6.9) | 0 |
| Flatulence | 2 (6.9) | 0 |
| Headache | 2 (6.9) | 0 |
| Neuropathy peripheral | 2 (6.9) | 0 |
| Photophobia | 2 (6.9) | 0 |
| Somnolence | 2 (6.9) | 0 |
| Thrombocytopenia | 2 (6.9) | 0 |
| Stomatitis | 2 ( 6.9) | 1 (3.4) |
| Leukopenia | 1 (3.4) | 1 (3.4) |
| Upper gastrointestinal haemorrhage | 1 (3.4) | 1 (3.4) |

**Table 3S.** Dose reductions and interruptions

| Action (N=29) | Investigator assessment |
| --- | --- |
| Dose reductions, N (n%)  0  1  ≥2 | 18 (62.1)  6 (20.7)  5 (17.2) |
| Number of dose reductions per patient, median (range) | 0 (0 - 8) |
| Reason for dose reductions, number of events  Adverse events  Clinical decision  Dosing error | 21  1  2 |
| Dose interruptions, N (n%)  0  1  ≥2 | 13 (44.8)  8 (27.6)  8 (27.6) |
| Number of dose interruptions per patient, median (range) | 1 (0 - 5) |
| Reason for dose interruptions, number of events  Adverse events  Clinical decision  Dosing error  Progressive disease | 24  3  9  1 |

**Table 4S.** Pharmacodynamic Results

|  | Phosphate | FGF23* | FGF19* | FGF21* |
| --- | --- | --- | --- | --- |
|  | mg/dL | pg/mL | pg/mL | pg/mL |
| Baseline | 3.1 | 25.8 | 158 | 742 |
| R_Max_ | 4.3 | 166 | 519 | 688 |
| BR_Max_ | 1.2 | 140 | 361.2 | -54 |
| %BR_Max_ | 38 | 544 | 229 | -7.2 |
|  | % | % | % | % |
| %DiffC2D1 | 37 | 208 | 204 | 5.6 |
| %DiffMax | 45 | 786 | 558 | 103 |

Rmax: Maximum observed response value

BRmax: Maximum change from baseline response value

%BRmax: BRmax maximum percent change from baseline (B) response value

%DiffC2D1: Average percentage difference from baseline on Day 1 Cycle 2

%DiffMax: Overall average percentage difference

***** For FGF19 and FGF21 patients 96, 99, 106, 109, 118 and 119 were excluded. For FGF23 patients 80, 96, 99, 106, 109, 118 and 119 were excluded.
